# Do Parasitic Trematode Cercariae Demonstrate a Preference for Susceptible Host Species?

**DOI:** 10.1371/journal.pone.0051012

**Published:** 2012-12-18

**Authors:** Brittany F. Sears, Andrea D. Schlunk, Jason R. Rohr

**Affiliations:** Department of Integrative Biology, University of South Florida, Tampa, Florida, United States of America; Centro de Pesquisa Rene Rachou/Fundação Oswaldo Cruz (Fiocruz-Minas), Brazil

## Abstract

Many parasites are motile and exhibit behavioural preferences for certain host species. Because hosts can vary in their susceptibility to infections, parasites might benefit from preferentially detecting and infecting the most susceptible host, but this mechanistic hypothesis for host-choice has rarely been tested. We evaluated whether cercariae (larval trematode parasites) prefer the most susceptible host species by simultaneously presenting cercariae with four species of tadpole hosts. Cercariae consistently preferred hosts in the following order: *Anaxyrus* ( = *Bufo*) *terrestris* (southern toad), *Hyla squirella* (squirrel tree frog), *Lithobates* (* = Rana*) *sphenocephala* (southern leopard frog), and *Osteopilus septentrionalis* (Cuban tree frog). These host species varied in susceptibility to cercariae in an order similar to their attractiveness with a correlation that approached significance. Host attractiveness to parasites also varied consistently and significantly among individuals within a host species. If heritable, this individual-level host variation would represent the raw material upon which selection could act, which could promote a Red Queen “arms race” between host cues and parasite detection of those cues. If, in general, motile parasites prefer to infect the most susceptible host species, this phenomenon could explain aggregated distributions of parasites among hosts and contribute to parasite transmission rates and the evolution of virulence. Parasite preferences for hosts belie the common assumption of disease models that parasites seek and infect hosts at random.

## Introduction

Parasitism is the most common consumption strategy among heterotrophs [1] and parasitic associations undeniably involve some of the most intimate co-adaptations between organisms. Generalist parasites, capable of infecting multiple host species, might possess adaptations that extend beyond single host-species interactions to include traits affording discrimination among host species. Indeed, generalist parasites frequently demonstrate behavioral preferences for particular host species when presented with multiple suitable host species. A number of characteristics can influence these preferences, including host abundance [2], reproductive status [3], or species identity [4], [5], [6]. Several proximate explanations have been offered for these preferences, such as reduced host vigilance against ectoparasites during reproduction [3] or parasite preference for a particular host species as the result of historical sympatry [2]. At the ultimate level, these preferences are presumed to be adaptive decisions that maximize parasite fitness, but it remains unclear and nearly un-investigated whether patterns of host selection by parasites are attributable to host competence. The competence of a host to a parasite can be influenced by several traits, including host survival post-infection and interactions between infected and uninfected hosts, but the first hurdle a parasite must surmount is to successfully infect a host, which requires a susceptible host. Given that hosts can vary widely in their susceptibility [7], an examination of whether generalist parasites infect the most competent hosts must begin with an examination of whether parasites select among hosts based on susceptibility to infection.

The larvae of digenetic trematodes, cercariae, are frequently generalists in their host use [8]. Cercariae actively swim through the water column, detecting hosts via physical and chemical cues [5], [9], [10], [11], [12], [13]. Within a species of host, susceptibility to a particular species of cercariae may depend on a number of factors, including host genotype, habitat, or infection history [14], [15], [16]. Given that 1) the reproductive success of cercariae depends on their ability to successfully infect a host, 2) cercariae can often infect a range of host species, and 3) use chemical cues to detect hosts, they might demonstrate a preference to infect the most susceptible hosts available.

**Figure 1:**
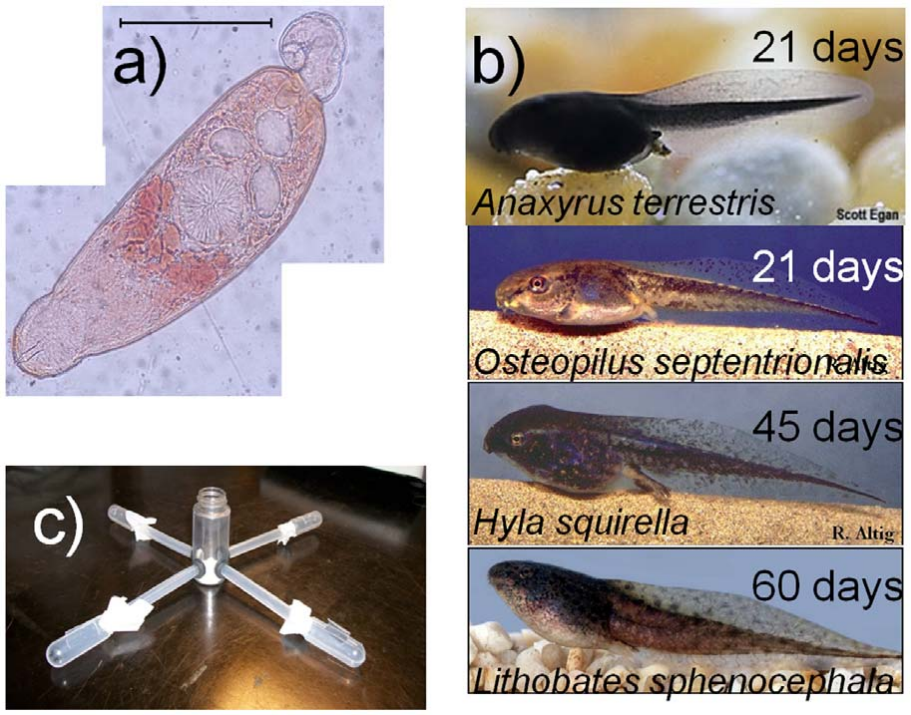
Focal species and experimental host-selection apparatus. **a)** Neutral red-stained focal parasite’s armatae cercaria; scale bar = 0.5 mm. **b)** four focal amphibian species with average larval period length (days from hatching to metamorphosis) in upper right corner. **c)** Host selection apparatus. Cercariae were placed in the center vial and one tadpole was placed inside of each of four pipette bulbs. Photo credit: a) Scott Egan, b–c) Ron Altig, d) United States Geological Survey.

In this experiment, we tested whether a cercarial species local to Tampa, FL (Fig. 1a) exhibits preferences for certain sympatric, simultaneously-presented tadpole species (Fig. 1b; *Anaxyrus terrestris, Hyla squirella, Lithobates sphenocephala,* and *Osteopilus septentrionalis*) and, in independent trials, whether their preference for particular host species correlates with susceptibility of hosts to infection. We predicted that among-individual variation in attractiveness and susceptibility to cercariae would be greater than within-individual variation in these traits. Furthermore, because *O. septentrionalis* has been recently introduced to this locality (post-1997; [17], [18]), we predicted that it would be the least-preferred host because cercariae are unlikely to have co-adapted to its cues.

## Materials and Methods

### Study System

Digenetic trematodes typically have a three-host lifecycle, including a molluscan first intermediate host. We collected *Planorbella trivolvis* snails in the Tampa, FL area in July of 2009 and screened them for trematode infections based on the presence or absence of free-swimming armatae cercariae (Fig. 1a). These cercariae infect tadpoles by directly penetrating the skin and encysting subcutaneously as metacercariae. If an infected tadpole is eaten by the definitive host, typically a vertebrate, the metacercariae develop into adult trematodes in the host’s intestine, from which it will shed eggs that hatch into miracidia, completing the life cycle when they infect new snail hosts.

Tadpoles used in this experiment were collected in August and September of 2009 from wetlands in the Tampa, FL area. Both snails and tadpoles were collected in accordance with permit WX08179 from the State of Florida Fish and Wildlife Conservation Commission. The species of tadpoles used here are frequently sympatric with trematode-infected *P. trivolvis,* but all tadpoles were collected from snail-free habitat and were collected soon after hatching and maintained in the lab free of trematodes until we all four species were collected for the trials. Hence, it is unlikely that any of the tadpoles were infected before exposure to cercariae in this experiment. When used in trials, tadpoles ranged in Gosner developmental stage [19] from stage 28–37; we included Gosner stage as a covariate in our analyses (see “Statistical Analyses,” below) to control for within-species differences in size and potential effects of Gosner stage on host immunology (e.g., [20], [21]). Tadpoles were maintained in artificial spring water [9] and fed frozen spinach *ad libitum* between experimental trials.

### Parasite Identification

The cercariae used in this experiment are an armatae morphotype, which occurs exclusively in the families Plagiorchiidae and Telorchiidae [22]. To identify cercariae more specifically, we sequenced DNA from the 18S region of armatae cercariae from snails collected at the same site as those used in this experiment. DNA from approximately 100 cercariae was extracted using an UltraClean® Fecal DNA kit (Mo-Bio Laboratories). The primers BD1/4S and 3S/ITS2.2 from Wilson et al. [23] were used to amplify the ITS-1 and ITS-2 regions. PCR was carried out in a total volume of 25 µl, which consisted of 1 µl of each primer (10 µM), 10 ng DNA, and 12.5 µl PCR Master Mix (Promega Corporation). Reaction conditions were identical to Wilson et al. [23]. PCR product was viewed on an ethidium bromide-stained 1% agarose gel. DNA sequencing was conducted by Eurofins MWG Operon. Sequence results indicated 100% sequence similarity to both *Renifer aniarum* and *Lechriorchis tygartii,* both of which belong to the subfamily Reniferinae in the family Plagiorchiidae [24]. Reniferin trematodes are fairly conserved in their host use, utilizing freshwater snails as first intermediate hosts, tadpoles as second intermediate hosts [25], [26], and snakes as definitive hosts [27], [28]. Given that the sequenced cercariae were a close match to both of the above species, it likely exhibits a similar life history as other reniferin species.

### Parasite Preference for Host Species

Selection chambers were used to determine whether cercariae demonstrated a behavioural preference among four species of tadpoles (Fig. 1c). The basic design was as follows: four 9.5 mm holes were drilled at 90° angles to one another into a 20 mL scintillation vial. To prevent water leakage, holes were fitted with rubber grommets, each of which held one horizontal transfer pipette. The tube and bulb of each transfer pipette were separated by Nitex mesh (11 µm), secured by silicone caulk. The Nitex mesh allowed tadpole chemical cues to pass, but prevented the cercariae from infecting the amphibians. The bulb of each transfer pipette was cut to create flaps, allowing space for a tadpole to be inserted. The size of the bulb prevented tadpoles from most movement. The apparatus was filled with 5 mL artificial spring water ASW, a quantity that reached just above the rubber grommets, which prevented cercariae from travelling vertically. Thus, there was a central region from which four arms projected, each holding a different tadpole species. No negative control trials (i.e., empty arms) were conducted.

We allowed tadpoles to condition the water with chemical cues for 10 minutes, forming a gradient, then added approximately 30 cercariae (range: 31–35) to the center of the scintillation vial. The cercariae were allowed to swim freely for 30 minutes. After 30 minutes, each tube was sealed off from the central scintillation vial using a metal clamp. Tadpoles were removed from the apparatus and returned to individual 1-L cups. The water and cercariae from each arm and the central scintillation vial were pipetted separately into petri dishes. Cercariae were killed and stained with Lugol’s iodine and counted at 40x magnification under a dissecting microscope. Cercariae which swam into a given arm were assumed to have selected the host at the distal end of the arm for infection.

Five selection chambers were run simultaneously on 10 days for a total of 50 trials over the course of the experiment. All trials were conducted under artificial, fluorescent light at 23°C and tadpoles were rotated among the chambers’ arms to prevent biased effects of any particular arm. All cercariae used were more than one hour old and less than 12 hours old, as cercariae have a <24-hour lifespan and several-hours-old cercariae are more infective than new or old cercariae [29]. Two trials were excluded from the analyses because only one and two cercariae, respectively, made choices in these trials. Individual tadpoles were repeat-tested between one and five times (average use: 2.5 trials per tadpole) throughout the 10 days of trials. This allowed us to evaluate whether among-individual variation in attractiveness to cercariae was greater than within-individual variation in attractiveness. To calculate the proportion of cercariae making a choice among hosts, the number of cercariae remaining in the center chamber was subtracted from the initial number added to the apparatus. The number of cercariae in each arm was then divided by the total number of cercariae that entered any arm.

### Susceptibility of Host Species

One week after the parasite preference trials were completed, we conducted a second experiment testing if host species differed in their susceptibility to parasites (i.e., the ability to prevent infection or limit the intensity of infection). The same tadpoles utilized in the parasite preference portion of the experiment (above) were isolated in individual plastic specimen cups with 25 ml of artificial spring water for experimental exposure to infective cercariae. The dose of infective cercariae was 20 cercariae for *A. terrestris* and 30 for *H. squirella, L. sphenocephala,* and *O. septentrionalis.* The differences in dose were due to variation inthe output of cercariae by infected snails on the day of exposure. All of the cercariae penetrated the tadpoles within 12 h (i.e., none were found alive or dead at the bottom of the cups). After 48 h, tadpoles were euthanized in a 0.01% benzocaine solution and preserved in 70% ethanol. Tadpoles were then demelanized and cleared in order to make parasite cysts visible, as detailed by [30]. In brief, 30% hydrogen peroxide was added in increments of 1% of the total ethanol volume until the tadpole was colourless; specimens were then cleared in a solution of 0.5% potassium hydroxide and glycerol until transparent. Metacercarial cyst quantification was performed under a compound light microscope at 100x. The majority of *L. sphenocephala* individuals used in the preference trials were damaged during the clearing protocol and, thus, their metacercariae could not be quantified. We subsequently experimentally infected other *L. sphenocephala* tadpoles to obtain susceptibility data, but lacked the susceptibility data from the same tadpoles used in preference trials with which to quantify the relationship between attractiveness and susceptibility.

### Statistical Analyses

For the cercarial choice data, we conducted an analysis of variance (ANOVA; type II sums of squares) on the arcsine square-root-transformed proportion of cercariae that selected each tadpole species in each trial. In this analysis, we blocked by each of the 10 days we conducted host preference trials, weighted the analysis by the number of cercariae that made a choice in each trial, and tested for an effect of tadpole species and individuals nested within tadpole species. Block, host species, and individuals were all treated as random effects.

For the susceptibility data, we conducted an ANOVA on the arcsine-square-root transformed proportion of cercariae that encysted, controlling for individual Gosner developmental stage [19] and testing for differences among species. Gosner stage and frog mass were correlated (*r* = 0.528, *P* = 0.036), Gosner stage is independent of water and fecal loss, and there are well-documented changes in susceptibility to trematodes with Gosner stage independent of mass [21], so we chose to present the results with Gosner stage as the covariate. However, the results did not qualitatively change when mass was substituted as the covariate. We conducted Fisher’s least significant difference posthoc multiple comparison tests to determine which host species significantly differed in their attractiveness and susceptibility to the cercariae. We used regression analysis (one-tailed) to determine whether there was a positive relationship between host susceptibility and attractiveness to cercariae using the species as the replicate. Both cercarial choice and susceptibility analyses were performed using Statistica software v9.1.210 [31].

## Results

### Parasite Preference for Host Species

**Figure 2:**
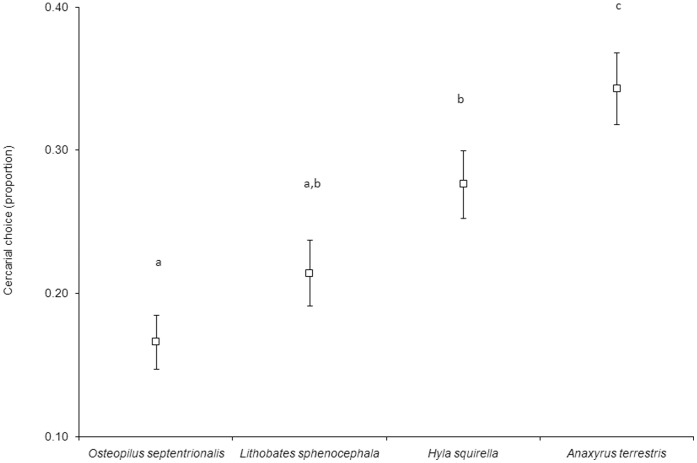
Proportion (mean ±1 SE) of experimentally-administered cercariae demonstrating a preference for a host species. Values for those species not sharing a letter are significantly different based on Fisher’s least significant difference tests.

Parasite preference for hosts significantly differed between host species (*F*_3,43.70_ = 5.27, *P = *0.003); parasites demonstrated the strongest preference for *A. terrestris*, followed by *H. squirella*, *L. sphenocephala*, and *O. septentrionalis* (Fig. 2). Among-species variation in host attractiveness to cercariae was greater than within-species variation in attractiveness, but individual identity within a species was also a significant predictor of attractiveness (*F*_61,118_ = 1.82, *P* = 0.003). That is, there was greater variation in attractiveness to cercariae among than within individuals of a species, but there were also significant and repeatable differences in the attractiveness of individual tadpoles.

### Susceptibility of Host Species

**Figure 3:**
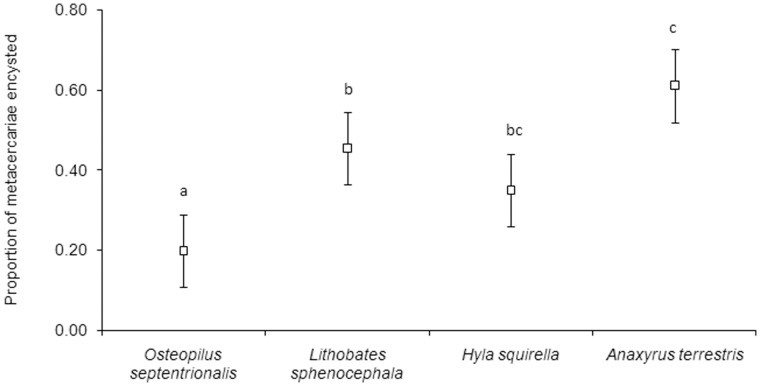
Proportion (mean ±1 SE) of experimentally-administered cercariae successfully infecting each host species. Values for those species not sharing a letter are significantly different based on Fisher’s least significant difference test.

Independent of developmental stage (*β* = −0.659, *F*_1,54_ = 24.92, *P<*0.001), host species also varied significantly in their susceptibility to infection (*F*_3,54_ = 7.55, *P<*0.001; Fig. 3), with *A. terrestris* being most susceptible, followed by *L. sphenocephala*, *H. squirella*, and *O. septentrionalis* (Fig. 3). The relationship between a species’ attractiveness to cercariae and its susceptibility to cercariae was positive and approached significance (*F*_1,1_ = 4.82; *P* = 0.08).

## Discussion

Cercariae discriminated among host species and tended to demonstrate a preference for the most susceptible host species but this relationship was not statistically significant. Tadpoles of *A. terrestris* were the most preferred and most susceptible to infection by cercariae, with *H. squirella, L. sphenocephala,* and *O. septentrionalis* exhibiting reduced attractiveness and susceptibility in the order listed (Figs. 2, 3). Although the tadpole species used here differ in size, the proximate driver of attractiveness is unlikely to be attributable to simple size differences. First, the most attractive species, *A. terrestris,* is also the smallest, so cues from *A. terrestris* were probably not more abundant than those of other species. At the individual level, size is again an unlikely explanation for attractiveness because we controlled for Gosner stage in our analyses, which typically correlates with size among individuals of a species. Nutritional differences are also unlikely to explain within-species differences in attractiveness and susceptibility because, although poor nutrition can retard growth and increase susceptibility to infections in tadpoles [32], individuals tested here were from the same location (within species) and were fed *ad libitum*. Finally, 93% (57/61) of the Cuban, Squirrel and Leopard frogs were below Gosner stage 42 and frogs don’t begin losing mass until after stage 42 (see [33]). The toads were all the same Gosner stage (i.e. no variability). Therefore, it is unlikely that mass or a mass-by-Gosner stage interaction influenced the outcome of the trials.

Life history differences, however, may partially explain the variation in susceptibility among species. Adults of species with a short larval period, such as *A. terrestris,* tend to lay their eggs in ephemeral water bodies, including ditches and puddles [34] that rarely support permanent snail populations (pers. obs.). The absence of snails, and therefore cercariae, might free tadpoles of the selective pressure necessary to maintain a robust immune response to trematodes, leading to increased susceptibility to parasitism. Indeed, other experiments have demonstrated that tadpole species with short larval periods are more susceptible to infection by cercariae than species with long larval periods [7], [35]. Furthermore, life history differences can also be coupled with behavioural differences (e.g., [36]), which might have contributed to the attractiveness of *A. terrestris*.

We included *O. septentrionalis* in this study to explore whether its success as an introduced species might be attributable to cercariae in its non-native range failing to exhibit a preference that is proportional to their susceptibility, perhaps because of a lack of co-evolutionary history. Such a pattern would contribute to a commonly observed phenomenon, in which introduced species have few parasites in their non-native range, formalized in the “enemy release” hypothesis [37]. The trematodes used in this experiment are likely novel to *O. septentrionalis;* the only trematode species described from *O. septentrionalis* in its native range is *Mesocoelium crossophorum* [38]. We can be confident that *M. crossophorum* is not the parasite used in this experiment because 1) it is in a different family (Brachycoeliidae [39]) than the species suggested by DNA sequencing and 2) the cercariae of *M. crossophorum* are dissimilar from those used here; most notably, cercariae of *M. crossophorum* are tailless [40], whereas ours possess a well-developed tail (Fig. 1a). *O. septentrionalis* was not significantly less attractive or more susceptible than every native species, but it ranked lowest among host species for both measures (Fig. 2,3). Future studies should investigate whether introduced species are less attractive to active, host-seeking parasites and vectors of parasites than native species and whether this contributes to enemy release.

In this experiment, individual hosts exhibited consistent variation in their attractiveness to cercariae. Consistent individual variation in a trait, in this case attractiveness to parasites, is the material upon which natural selection acts. If such a trait is heritable, then one might expect a Red Queen-type evolutionary arms race between host evasion of parasites through altered chemical cues and parasite counter-adaptations to remain capable of identifying and infecting the most susceptible hosts. The specific cues used by cercariae to locate tadpoles are not clear, but host peptide cues are frequently utilized by echinostomatid cercariae [41]. The chemical composition of tadpole skin might have wide variation, given interspecific differences in traits such as palatability [42], which is presumably governed by chemicals such as toxins in the skin [43]. If variation in cercarial host preference and resistance does correlate with tadpole susceptibility and length of larval period, as detailed above, parasites should constitute a selective pressure affecting tadpole life history evolution at the ultimate level, as well as expression of chemical cues at the proximate level [44], [45].

In summary, our results suggest that, among native host species, cercariae seem to select the most susceptible host species and exhibit consistent preferences for certain individuals within a species. If this occurs commonly in nature, epidemiological models could be underestimating parasite transmission rates for parasites which infect multiple host species (but see [46]). Non-random host usage could explain patterns of parasite aggregation among and within host species [47], [48] and could be especially profound for those parasites that can actively seek out susceptible hosts. Effects of non-random host choice could also apply to parasites that manipulate vectors into seeking preferential hosts (see [44]). Given the role of infectious diseases in current amphibian declines [49], [50], adjusting these models could be crucial for accurately predicting and mitigating amphibian declines.
